# Open peer review at four STEM journals: an observational overview

**DOI:** 10.12688/f1000research.6005.2

**Published:** 2015-07-20

**Authors:** Emily Ford

**Affiliations:** 1 Urban & Public Affairs Librarian, Portland State University Library, 1875 SW Park, Portland, OR, 97207, USA

**Keywords:** open peer review, peer review, scholarly communication, science communication, scholarly publishing

## Abstract

Open peer review, peer review where authors' and reviewers' identities are disclosed to one another, is a growing trend in scholarly publishing. Through observation of four journals in STEM disciplines, *PLOS One*, *Atmospheric Chemistry & Physics*, *PeerJ*, and *F1000Research*, an observational overview is conducted. The overview relies on defined characteristics of open peer review. Results show that despite differing open peer review implementations, each journal retains editorial involvement in scholarly publishing. Further, the analysis shows that only one of these implementations is fully transparent in its peer review and decision making process. Finally, the overview contends that journals should clearly outline peer review and editorial processes in order to allow for open peer review to be better understood and adopted by authors, reviewers, editors, and readers of science communications.

## Introduction

In scholarly publishing open peer review (OPR) is an emerging form of peer review that incorporates disclosure of author and referee identities to one another. Although in its infancy, OPR has been adopted and implemented in a number of disciplines and their respective scholarly publications. In this article I provide some background on OPR, addressing controversies and divergent opinions. Next I describe, examine, and discuss OPR implementations at four different science, technology, engineering, and mathematics (STEM) journals: *PLOS ONE*, *Atmospheric Chemistry & Physics*, *PeerJ*, and *F1000Research*. These observations contribute to our understanding of scholarly publication and scientific communication, as we watch the evolution of scientific vetting and validity determination processes.

## Open Peer Review: a definition

Unlike double-blind peer review, which is clearly defined, has clear parameters, and an arguably universal understanding by the scholarly community in how to implement it, OPR is approached and implemented in a variety of ways. There is no one universally accepted definition of OPR, which complicates investigations of its practices. As such, I rely on my previous definition, which broadly understands OPR as any scholarly review mechanism providing disclosure of author and referee identities to one another at any point during the peer review or publication process ( Ford, 2013). This definition is used as a starting point via which to observe OPR processes, and further analyze differing OPR implementations.

Other terms used to discuss OPR are peer-to-peer review and open review. Both these phrases insinuate OPR, but some have approached it as supplementary to formal peer review processes. For example, these review implementations rely on a community’s members to post comments on articles at pre-print servers, such as arXiv, or using comment features via journal websites, such as *British Medical Journal* and *BioMed Central*. It should be noted that when I mention OPR, I discuss it as the formal process via which scholarly articles are vetted for publication.

Mentions of OPR in scholarly literature date back to Michael McGiffert’s 1988 article, “Is Justice Blind? An Inquiry into Peer Review”, in which McGiffert argues, based on survey results, that editors should protect the identity of authors, but that editors, “...should leave referees free to decide for themselves whether or not to make themselves known [to the author]” (p. 47, McGiffert, 1988). Over time attitudes toward OPR have evolved and support of OPR has grown, although it still remains debated. Although OPR is a phenomenon occurring across the academic disciplines, those in STEM are the most prolific. Perhaps the oldest implementation of OPR occurred at *Atmospheric Chemistry and Physics* with its launch in 2001, which is discussed later in this article.

## Why Open Peer Review?

For many OPR addresses inherent issues in what has been the gold standard of double-blind peer review. Some see blind review processes as faulty in that referee anonymity allows for referee abuse. Others view OPR as a means to hold referees and authors accountable for their communications ( Cope & Kalantzis, 2009; Fitzpatrick, 2010; Mulligan, 2008). It has also been argued that OPR allows for easier identification of scientific misconduct ( Boldt, 2011), and that over time the quality of submitted articles will improve ( Hu *et al.*, 2010; Prug, 2010). OPR affords referees the ability to gain credit for and cite their contributions to science communication ( Boldt, 2011; Bornmann & Daniel, 2010; Fitzpatrick, 2010; Prug, 2010; Pöschl, 2009). More broadly speaking, some OPR implementations provide the scholarly community an insight into author/referee conversations during the review process. Surfacing these conversations provides readers an expanded contextual discussion of the subject at hand, and enriches science communication for all stakeholders ( Fitzpatrick, 2010; Friedman *et al.*, 2010; Lipworth & Kerridge, 2011; Maharg & Duncan, 2007). Finally, perhaps the most convincing pro argument for OPR asserts that OPR processes allow for quicker publication and dissemination of scientific findings ( Cope & Kalantzis, 2009; Hu *et al.*, 2010; Pöschl, 2004).

One of the major arguments against OPR is the perceived protection afforded both authors and reviewers in a blind process. For junior researchers serving as reviewers, blind review may allow them to feel more able to provide honest constructive criticism to senior researchers. Similarly, as authors, blind review is perceived as protecting junior researchers from public humiliation ( Godlee, 2003). It has also been noted that some reviewers refuse to participate in OPR implementations, or still have concerns about them ( Janowicz & Hitzler, 2012). These concerns still pervade conversations about OPR. Most recently, a survey of *BMC Pharmacology and Toxicology* Editorial Board Members surfaced continuing concerns regarding OPR at the journal. Despite these concerns *BMC Pharmacology and Toxicology* decided to “...continue with open peer review at *BMC Pharmacology and Toxicology* because of the ethical grounds for doing so and because the potential benefits outweigh the negatives”, (p. 4, Moylan *et al.*, 2014). This is evidence that despite continuing concerns and resistance to it, OPR will continue to be implemented and evolve in STEM publishing. As such, scholars should understand OPR implementations in order to further innovate and evolve scholarly publishing and scientific communication.

## Methods

Four STEM journals claiming to use OPR processes were chosen for observation. These four journals were selected because they represent a difference in relative age, their perceived stature or authority in STEM, and for the salience of information regarding OPR on their respective websites. To review and understand the four different peer review implementations, this observation relied on the eight OPR characteristics I identified in 2013. It should be noted that I relied on these characteristics because there is no other documented common vocabulary used to discuss and analyze open peer review. I do not argue, however, that each characteristic carries the same weight and influence on review. Rather, publishers of publications exhibiting these characteristics may weigh their resulting reviews different in publishing decisions. The characteristics are cited in full below:

- Signed review refers to submitted reviews signed by the referee that are either published alongside articles at the time of publication or are signed when an author receives them.
- Disclosed review refers to a process in which referees and authors know each others’ identities during the peer review process, enabling them to engage in discussion or discourse.
- Editor-mediated review is a characteristic found in most open peer review processes. Editor mediation is any work done by a journal editor to facilitate open peer review. This may include editorial preselection of articles and/or final decision-making for acceptance or rejection of articles. The editor-mediated portion of any open peer review process may or may not be publicly disclosed.
- Transparent review refers to complete openness to a distinct community or the public. It allows a public community to watch peer review unfold. Authors and the public know referees’ identities, and referees know authors’ identities. Author responses to referee comments are public. In transparent review the public can see manuscripts, reviews, and replies from authors and public reviewers as well as the published articles.
- Crowd-sourced review is a public review process in which any community member may contribute to the article review. In crowd-sourced review there is no limit to the number of comments or reviews an article may receive. In some proposed implementations of crowd-sourced review, there is little editorial mediation of article reviews. Rather, authors may simply submit papers to a preprint server or other community for crowd-sourced commentary.
- Pre-publication review occurs prior to article publication, and typically occurs in a public space such as a pre-print server.
- Synchronous review occurs at the same time as publication of the article. In the literature, synchronous review is approached only theoretically, as part of a novel and completely iterative publishing model.
- Post-publication review occurs after an article is published, much like commentary on a blog post (pp. 314–315, Ford, 2013).

Using these characteristics I examined information for authors and about each publication at their respective websites, promotional materials, blogs, and other materials discussing the OPR processes at each journal. Data for these observations was gathered in mid to late 2013. Publisher/journal policies and practices may have since changed.

## The Journals

### *PLOS ONE* 

*PLOS ONE* is an international publication of Public Library of Science, a not-for-profit publisher and open access advocacy organization. The journal was formed around the philosophy and practice that all research using scientifically sound research methods should be published regardless of its results, novelty, and/or impact. The journal publishes research articles from science and medical disciplines, including those reporting negative results. By publishing research from multiple disciplines, the journal asserts “ *PLOS ONE* facilitates the discovery of the connections between papers whether within or between disciplines”.

*PLOS ONE* launched in December 2006 and has since seen tremendous growth. It is indexed in numerous databases; is frequently cited as a source of research in news and popular media; and has received positive press for its review process. Even John Bohannon, a science journalist who undertook a sting operation of open access journals in an attempt to uncover poor publishing practices, acknowledged the strength of *PLOS ONE’s* review process ( Bohannon, 2013, ¶ 9).

All articles published in *PLOS ONE* carry Creative Commons attribution licenses. Although most authors publishing in the journal pay article processing charges (APCs), the journal makes exceptions and waives publishing fees for unfunded research. Moreover, the APC fee model at *PLOS ONE* takes into account an author’s country of origin, and whether it is a high, lower middle, or low income nation. In this way the publication aims to make more viable open access publication for authors with disparate economic means.

Compared to the other journals I discuss in this article, *PLOS ONE* conservatively approaches OPR. The journal’s peer review process only exhibits a few OPR characteristics, and even then these characteristics are not consistently implemented. It does, however, always use a form of editor-mediation for reviewing and publishing its content. Each submitted article is assigned an Academic Editor, who then determines whether submissions should be considered for peer review, and who facilitates the peer review process. According to the journal’s review guidelines, these Academic Editors may, “...conduct the peer review themselves, based on their own knowledge and experience; they can take further advice through discussion with other members of the editorial board; they can solicit reports from further referees”.

While the editorial mediation of journal articles at *PLOS ONE* always occurs, other characteristics of OPR do not. Signed reviews are optional, but they are strongly encouraged. According to its peer review guidelines, “If Peer Reviewers are willing, then they are also identified to the author at the time of decision.” *PLOS ONE* does not post reviewer comments to the web alongside published articles, so readers do not benefit from reading discussions that occurred about the topic prior to publication. In addition to *PLOS ONE’s* version of signed review, the journal enables a public commentary function for published articles. Although this technical functionality could be considered crowd-sourced reviewing, the journal does not consider post-publication public comments and discussions as part of a formal peer review process. One aspect of this crowd-sourced discussion process is that *PLOS ONE* surfaces any media coverage of published articles by linking to them in an article’s comments section. The result is that the journal is able to create a record of the impact and conversation an article elicits outside of the *PLOS ONE* platform and community.

### *Atmospheric Chemistry and Physics* 

Perhaps the oldest of the open peer reviewed publications is *Atmospheric Chemistry and Physics* ( *ACP*), the European Geosciences Union’s journal. The journal, which launched in September of 2001 ( Pöschl, 2004), publishes research articles, review articles, technical notes, peer-reviewed comments, correigenda, and supplementary materials. All published content is published under a creative commons 3.0 attribution license and authors are subject to APCs.

Following an article’s submission to *ACP* and brief editorial review, it is then hosted on the journal’s pre-print server, *Atmospheric Chemistry and Physics Discussion Papers* ( *ACPD*) for peer review and crowd-sourced discussion. On this platform reviewer comments and crowd-sourced comments are publicly available. After the discussion period for a paper ends, the ability to comment on the paper is turned off, and the journal editor makes a final publishing decision using submitted referee and public reviews. When the editor accepts an article for publication, the article is published at *ACP*, where it will also link to its pre-print version including referee and public comments at *ACPD*. *ACP* does not host public commentary on published articles. Although *ACP* views its peer review process as completely transparent ( Pöschl, 2004), it is not. Reviewers may choose to disclose their identities, or they may choose to remain anonymous. True transparency of the review process can only occur when reviewers and public commenters have disclosed their identities.

In addition to editor mediation and crowd-sourced reviewing, *ACP’s* process employs disclosed review and pre-publication review. It could be argued that synchronous review occurs, yet *ACP* does not consider papers posted at *ACPD* to be “published”. Because these papers are not considered published, their review is not synchronous.

### *PeerJ* 

At the time data was collected for this article, PeerJ was an individual membership-based publisher in the biological and medical sciences. However, during the review process for this article, a reviewer surfaced that PeerJ has since changed its model and now views it as a publication plan, rather than an individual membership ( Binfield, 2015). All works published by PeerJ are licensed with a Creative Commons Attribution 4.0 license. Its main publication, *PeerJ,* only publishes research articles. All other publication types are referred to *PeerJ PrePrints*, its pre-print repository and publication. *PeerJ* is young. The publisher first announced its publication model in June 2012, and published its first article on February 13, 2013. Its model relies on membership, where individuals pay one fee to PeerJ and become lifetime members. Based on an individual’s membership level—basic, enhanced, or investigator—individuals may publish in *PeerJ* a specified number of times per year.

The publishing model at *PeerJ* is similar to the pre-print/publication relationship between *ACP* and *ACPD* in that it maintains two publishing platforms: *PeerJ* and *PeerJ PrePrints*. Arguably, *PeerJ PrePrints* is not a publication, but a pre-print repository service. However, unlike *ACP*, *PeerJ* considers *PeerJ PrePrints* a publication, so I will treat it as such alongside *PeerJ*.

Only paid PeerJ members may publish work in *PeerJ*. Works may first be submitted to *PeerJ PrePrints*, or may be directly submitted to *PeerJ*. *PeerJ’s* article review, acceptance and publication model mirrors *PLOS ONE’s*; *PeerJ* accepts scientifically sound research and does not consider an article’s “novelty, interest, or impact” as part of its editorial criteria. Prior to review, submissions to *PeerJ* undergo editorial vetting by assigned Academic Editors. These Academic Editors are responsible for facilitating peer review of assigned articles to be completed by at least two reviewers, and making final publication decisions. Additionally, Academic Editors are attributed alongside each published article. Authors and reviewers alike are “encouraged” to post the full peer review cycle online alongside final versions of articles, but the journal does not mandate it. Based on information from *PeerJ’s* website, it is unclear whether author and reviewer identities are disclosed to one another during the review process. However, during review of this article, a reviewer clarified that *PeerJ* authors receive referee identity disclosure (if disclosed) at the time they receive their referee reports and publication decision from the Academic Editor ( Binfield, 2015).

Unlike the crowd-sourced review occurring at *ACP*, *PeerJ* is similar to *PLOS ONE* in that it views discussions and commentary on articles as separate from the formal peer review process. Unique to *PeerJ*, too, is its utilization of a broader discussion model named Q&A. *PeerJ’s* Q&A incorporates not only community questions and answers regarding pre-prints and articles—which can be posed at the paragraph and figure level—it also allows for free-standing questions and discussions of PeerJ community members. In this way, Q&A is intended to be a platform for anyone in the community to participate in scientific conversation. At *PeerJ* Q&A includes an incentive system for individual community participants. Contributing individuals are awarded points for their engagement. For example, one earns 100 points for authoring an article, 35 points for contributing an open review, etc. These points are displayed on members’ profile pages.

PeerJ has future plans to expand *PeerJ PrePrints*. The publisher hopes to allow authors to share as much or as little of their publications as they wish. They may decide to openly publish a title, title and abstract, or the whole paper. Additionally, *PeerJ PrePrints* will allow for authors to share papers “privately” with only particular users, only the PeerJ community, or fully open on the web.

### *F1000Research* 

Finally, the fourth of the journals I discuss, *F1000Research*, most consistently exhibits OPR characteristics. It is an open access journal published by Faculty of 1000. The publisher calls it one of “...four unique services that support and inform the work of life scientists and clinicians” provided by the publisher. *F1000Research* published its first approved article in July 2012, only six months after Faculty of 1000 announced the new publication. For published data the journal utilizes a Creative Commons No Rights Reserved license; it requests attribution for works, but anyone anywhere is free to use, build upon, and manipulate works. For published articles the journal uses a Creative Commons Attribution license. The journal itself includes case reports, clinical practice articles, commentary, correspondence, data articles, method articles, opinion articles, research articles, reviews, short research articles, study protocols, systematic reviews, thought experiments, and web tools in the Life Sciences. Authors submitting articles to *F1000Research* pay APCs.

At *F1000Research* articles undergo a peer review process after they are published. As such, *F1000Research* is the only publication I discuss that uses a post-publication review process. In this way the journal is able to speed up publication timelines to disseminate scholarly work; the journal publishes articles within one week of submission. As stated in its referee guidelines, the journal publishes submitted articles that pass initial editorial review for “content, quality, tone and format” as well as completeness, plagiarism, ethical standards, and adherence to author guidelines. In addition to reviews provided by two or three designated expert referees—which are attributed to the reviewer and are published online with the work—the scientific public (those affiliated with scientific or medical organizations) may comment on any published article. Any author responses to referees are also public.

In this publication model it is possible for articles to receive unanimous negative reviews. In this case, articles remain “published”, but are removed from the site’s default search. The site’s interface clearly delineates a work’s referee status and comments using icons to indicate: approved with reservations, approved, or not approved. It is important to note that *F1000Research* does not consider these statuses as equivalent to the accepted, accepted with revisions, and rejected statuses that one sees in closed review processes. Rather, as stated in the FAQs:

- The term Approved means that the referee thinks that the article is good and has either no suggested revisions or only minor revisions. The term Approved with Reservations means that the referee agrees that the article has scientific merit and is fundamentally sound but would like the author to make further changes to the manuscript. This is approximately equivalent to a request for major revisions or several minor revisions in a traditional journal. In every case, even when all referees approve of the article, future versions are welcome.

Because *F1000Research* publishes and attributes all referee responses and author comments, it adheres to a fully transparent peer review process. In addition to transparent reviews provided by pre-selected referees, crowd-sourced review occurs when individuals comment on published articles. The journal exhibits most other OPR characteristics; its reviews are editor-mediated, transparent, referee and author identities are disclosed, and reviews are signed. The only OPR characteristics not exhibited by *F1000Research’s* process are those related to review timing. Since all review at *F1000Research* occurs post-publication, the journal does not exhibit pre-publication and synchronous review characteristics. Once a work has been vetted by editors and is made public on the journal’s site, the journal considers it published. Because review occurs post publication, authors receiving critical feedback are encouraged to revise and submit updated versions of articles that will, again, be refereed. The journal uses CrossRef’s CrossMark identification service to assist readers in tracking these article versions and relationships. Even if an author publishes an updated article, previous versions remain published. In this way, publication at *F1000Research* is a good example of the iterative process of publishing and scientific knowledge and conversation.

## Discussion

I examined four journals using relatively OPR processes, *PLOS ONE*, *ACP*, *PeerJ*, and *F1000Research*. None of these journals have implemented OPR in the same manner, but they do exhibit many of the same OPR characteristics. Table 1 offers a comparison of the journals’ OPR characteristics, and points to a relative degree of just how open are their OPR processes.

**Table 1.** Open Peer Review Characteristics Comparison.

	*PLOS ONE*	*Atmospheric Chemistry &* *Physics*	*PeerJ*	*F1000Research*
Signed	Optional but strongly encouraged	Optional but strongly encouraged	Optional but strongly encouraged	Yes
Disclosed	Only if reviews are signed.	Only if reviews are signed.	Only if reviews are signed.	Yes
Editor-mediated	Always	Always	Always	Always
Transparent	No. Referee comments and author responses are not public.	Only if reviews are signed.	Only if reviews are signed and both referees and authors opt to share all comments generated during the review process.	Yes
Crowd-sourced	No. Reader comments are not considered part of the peer review process.	Yes	No. Reader comments are not considered part of the peer review process.	Yes
Pre-publication	Yes	Yes	Yes	No
Post-publication	No. Reader comments contributed to published articles are not considered a part of the peer review process.	No	No, though reader comments and the Q&A community may continue discussions regarding articles.	Yes
Synchronous	No	No	Possibly, since *PeerJ* *PrePrints* is considered a publication.	No. Articles are considered published once they are posted to the site.

Each journal exhibits a form of editor-mediation and each journal vets submitted articles prior to publication for basic quality, scope and adherence to author guidelines. While for some journals, such as *F1000Research*, this is as far as an editor’s work goes before an article is published, others, such as *ACP* and *PeerJ*, allow editors to make final publishing decisions.

Each of the journals allow for some form of crowd-sourced review. At *PeerJ* the Q&A section includes commentary on articles; it also includes other avenues for the public to engage in scientific conversations. At *PLOS ONE*, however, commentary on articles occurs only after an article has been published, and also includes links to all media coverage of articles. Unlike *PLOS ONE*, *PeerJ*, and *F1000Research’s* crowd-sourcing implementations, *ACP’s* process only allows for crowd-sourced commentary prior to publication articles during the discussion phase of the OPR process. Crowd-sourced review, although a characteristic of OPR, may be weighed differently in a publication’s review process ( Perakakis, 2015).

Another commonality between these publications is their varying allowance, encouragement, or mandate for reviewers to sign their commentaries. Referees may choose to remain anonymous at *ACP*, *PeerJ,* and *PLOS ONE* (even though disclosure is strongly encouraged), whereas *F1000Resarch PLOS ONE* requires referees to disclose their identities. Since author/reviewer identity disclosure is a defining factor of OPR for the purposes of this overview, it could be argued that those articles where reviewer commentary is not attributed are not truly open peer-reviewed. The motivation for publishers to encourage rather than require openness most likely stems from their desire to encourage more authors and reviewers to participate in alternative peer review processes. Publishers may also be attempting to be mindful of different discipline’s accepted publishing practices. In my own view, by not mandating public attributed review, publishers are weakening the power of OPR. However, incremental steps in OPR implementation are necessary to encourage participation and to move OPR to a completely transparent standard in the future.

Two of the journals discussed above include article pre-print mechanisms. Pre-print servers and mechanisms introduce confusion into understanding publishing and OPR. Just when is something that is open to be read on the web considered “published?” Where *ACP* does not consider papers posted to its pre-print space, *ACPD*, as “published”, *PeerJ PrePrints* is considered by its publisher a publication. Publishers’ definitions of when a scholarly work is “published” will continue to evolve as OPR processes evolve. It is unlikely that any definition will be uniformly held by all scholarly publishers.

Of the four publishers discussed, I maintain that *F1000Research* exhibits what we should consider the gold standard of transparent and OPR processes. The publication’s process is completely transparent; it publishes all commentary with attribution and makes salient referee decisions. Moreover, the mechanism it uses to track and correlate article versions and updates enhances and opens scholarly conversations. Yet, *F1000Research* maintains its editorial voice via editor-mediation prior to an article’s publication and by suppressing from search results articles receiving unanimous negative reviews.

Finally, it should be noted that all four publications discussed in this article are open access publications. It is logical that open access journals are more open to the idea of experimenting with peer review processes, since they already embrace the ethos of openness in their publication models. It is possible that there are non open access journals using OPR, but I am not aware of any of these publications. In fact, I surmise that very few authors and reviewers who choose to write and review for non open access journals instead of open access journals, would resist implementations of OPR.

## Conclusion

Scholarly journals are beginning to challenge traditional peer review practices by implementing OPR, yet each OPR implementation differs. By observing four different implementations of OPR I conclude that few OPR journals implement truly transparent review, yet each implementation values editorial work. Further, I maintain that distinguishing between publicly available preprints and publicly available published articles unnecessarily muddies the waters in understanding OPR. As OPR implementations proliferate, it is pertinent for journals to clearly outline any peer review process so that readers, authors, and reviewers can fully understand peer review implementations, decision making processes, and to provide for editorial transparency.

Future research is needed in a number of areas. First, we need to understand how scholarly communities and publishers define an article’s publication status. The fact that content appears readily accessible via the web does not mean publishers consider articles “published.” This inherent tension, based in a print publishing paradigm, will continue to introduce confusion to those searching for and reading scholarly research and writings on the web. Additionally, OPR occurring in non-open access journals should be investigated, as should OPR implementations in non-STEM journals.

## References

[ref-1] Binfield P: Referee Report For: Open peer review at four STEM journals: an observational overview [v1; ref status: indexed, http://f1000r.es/4yi]. *F1000Res.* 2015;4:6 10.5256/f1000research.6426.r7269 25767695 PMC4350441

[ref-2] Bohannon J: Who’s afraid of peer review? *Science.* 2013;342(6154):60–65. 10.1126/science.342.6154.60 24092725

[ref-3] Boldt A: Extending ArXiv.org to Achieve Open Peer Review and Publishing. *J scholarly Publ.* 2011;42(2):238–242. 10.3138/jsp.42.2.238

[ref-4] Bornmann L Daniel HD: Reliability of reviewers’ ratings when using public peer review: a case study. *Learn Publ.* 2010;23(2):124–131. 10.1087/20100207

[ref-5] Cope WW Kalantzis M: Signs of epistemic disruption: Transformations in the knowledge system of the academic journal. *First Monday.* 2009;14(4–6). 10.5210/fm.v14i4.2309

[ref-6] Fitzpatrick K: Peer-to-peer Review and the Future of Scholarly Authority. *Social Epistemology.* 2010;24(3):161–179. 10.1080/02691728.2010.498929

[ref-7] Ford E: Defining and Characterizing Open Peer Review: A Review of the Literature. *J scholarly Publ.* 2013;44(4):311–326. 10.3138/jsp.44-4-001

[ref-8] Friedman R Whitworth B Brownstein M: Realizing the Power of Extelligence: A New Business Model for Academic Publishing. *Int J Technol Knol Soc.* 2010;6(2):105–117. Reference Source

[ref-9] Godlee F: Peer Review in Health Sciences (2nd ed.). London: BMJ Books.2003 Reference Source

[ref-10] Hu C Zhang Y Chen G: Exploring a New Model for Preprint Server: A Case Study of CSPO. *J Acad Libr.* 2010;36(3):257–262. 10.1016/j.acalib.2010.03.010

[ref-11] Janowicz K Hitzler P: Open and transparent: the review process of the Semantic Web journal. *Learn Publ.* 2012;25(1):48–55. 10.1087/20120107

[ref-12] Lipworth W Kerridge IH Carter SM: Should Biomedical Publishing Be “Opened Up”? Toward a Values-Based Peer-Review Process. *J Bioeth Inq.* 2011;8(3):267–280. 10.1007/s11673-011-9312-4

[ref-13] Maharg P Duncan N: Black Box, Pandora’s Box or Virtual Toolbox? An Experiment in a Journal’s Transparent Peer Review on the Web. *Int Rev Law Comput Tech.* 2007;21(2):109–128. 10.1080/13600860701492104

[ref-14] McGiffert M: Is Justice Blind? An Inquiry into Peer Review. *Scholarly Publishing.* 1988;20(1):43–48.

[ref-15] Moylan EC Harold S O’Neill C: Open, single-blind, double-blind: which peer review process do you prefer? *BMC Pharmacol Toxicol.* 2014;15(1):55. 10.1186/2050-6511-15-55 25266119 PMC4191873

[ref-16] Mulligan A: Quality, certification and peer review. *Information Services & Use.* 2008;28(3–4):pp. 197–214. Reference Source

[ref-17] Perakakis P: Referee Report For: Open peer review at four STEM journals: an observational overview [v1; ref status: indexed, http://f1000r.es/4yi]. *F1000Res.* 2015;4:6 10.5256/f1000research.6426.r7267 25767695 PMC4350441

[ref-18] Pöschl U: Interactive journal concept for improved scientific publishing and quality assurance. *Learn Publ.* 2004;17(2):105–113. 10.1087/095315104322958481

[ref-19] Pöschl U: Interactive Open Access Peer Review: The Atmospheric Chemistry and Physics Model. *Against the Grain.* 2009;21(3):26–32. Reference Source

[ref-20] Prug T: Open-process academic publishing. *Ephemera: Theory & Politics in Organization.* 2010;10(1):40–63. Reference Source

