# Supplementary material for: Open peer review at four STEM journals: an observational overview
Source: F1000Res. 2015 Jul 20;4:6. Originally published 2015 Jan 9. [Version 2] doi: 10.12688/f1000research.6005.2 (PMC4350441; doi:10.12688/f1000research.6005.2)
Supplement: Peer review history

# Peer review: Referee response for version 2

**DOI:** 10.5256/f1000research.7309.r9576

**Published:** 2015-08-10

**Authors:** Pandelis Perakakis

**Affiliations:** 1 Department of Psychology, University of Granada, Granada, Spain

The issues raised in the first round of reports were not addressed and I therefore decide not to change the status of my initial assessment.

I have read this submission. I believe that I have an appropriate level of expertise to confirm that it is of an acceptable scientific standard, however I have significant reservations, as outlined above.

# Peer review: Referee response for version 2

**DOI:** 10.5256/f1000research.7309.r9575

**Published:** 2015-07-29

**Authors:** Peter Binfield

**Affiliations:** 1 PeerJ, San Francisco, CA, USA

No further comments

I have read this submission. I believe that I have an appropriate level of expertise to confirm that it is of an acceptable scientific standard.

# Peer review: Referee response for version 1

**DOI:** 10.5256/f1000research.6426.r7267

**Published:** 2015-03-04

**Authors:** Pandelis Perakakis

**Affiliations:** 1 Department of Psychology, University of Granada, Granada, Spain

Thank you for this interesting observation article and for the invitation to contribute my open peer review.

In the beginning, the article tries to disambiguate different “flavours" or implementations of open peer review (OPR) by identifying a number of OPR characteristics. Regarding this effort I would like to note the following issues that I think should be somehow addressed in a future version of the article:

- As already noted by Peter Binfield, the definition offered is limited to only one of the identified OPR characteristics, namely whether the reviews are signed or not.
- It is not clear what is the difference between reviews “signed when the author receives them” and  disclosed reviews. It seems that in both cases the names of authors and reviewers are disclosed from the beginning of the review process.
- Is disclosure of the reviewer’s identity necessary to initiate a discussion between authors and reviewers? Perhaps what you refer to here is an infrastructure to allow multiple iterations during the review process, as provided for example by Frontiers. This is different from simply revealing the name of the reviewer.
- There is a clear distinction between an unsolicited comment on an article and a formal review process by expert peers that receive a review invitation either by an experienced editor or the authors themselves.
- What is the alternative to editor mediation in peer review, if we consider that unsolicited commentaries do not constitute formal peer reviews? Is there another model where formal peer review is not editor-mediated? I know we proposed such model that we called author-guided peer review in *Perakakis, P., Taylor, M., Mazza, M., Trachana, V. (2010). Natural selection of academic papers. Scientometrics, 85 (2), pp. 553–559*, but I am not aware of any implementations. I repeat that I do not think we should consider crowd-sourced comments as equal to formal peer reviews.

And some other comments to consider:

- PLOS ONE clearly does not qualify as an OPR journal by any standards. Instead, I would be more interested to see a discussion on the implementation of OPR by Frontiers.
- I suspect there is a misunderstanding around the terms double-blind and single-blind review. The standard in most journals and disciplines is single-blind review where authors are blind to the name of the reviewer, but reviewers know the name of the authors. Double-blind refers to the process where neither authors nor reviewers are aware about the identity of each other.
- It is not entirely correct that PLOS ONE “waives publishing fees for unfunded research”. What they do is ask for proofs that authors cannot cover the APCs even from their own budget, sometimes even asking for personal financial records, which by the way I find unacceptable. Proving that there is no formal funding to publish the particular research is not enough to waive APCs.
- I definitely agree that a comparison table is needed.
- I detected that the references to the articles by Lawrence are missing. Please double check that all references are included.

Thank you again for the opportunity to participate in this process that is very close to my ideal model of OPR and I will be looking forward to a revised version of your manuscript.

I have read this submission. I believe that I have an appropriate level of expertise to confirm that it is of an acceptable scientific standard, however I have significant reservations, as outlined above.

# Peer review: Referee response for version 1

**DOI:** 10.5256/f1000research.6426.r7273

**Published:** 2015-03-03

**Authors:** Ulrich Pöschl

**Affiliations:** 1 Multiphase Chemistry Department, Max Planck Institute for Chemistry, Mainz, Germany

Thanks to the author for an interesting perspective article on open peer review. Following an invitation to review this article, I would like to request a couple of specific corrections and add a general comment and suggestion.

1. Correction of Erroneous Statement: "Although *ACP* views its peer review process as completely transparent ( Pöschl, 2004), it is not."
  Neither the cited reference nor the ACP journal web pages claim that the peer review process of ACP would be "completely transparent". There are good reasons to maintain an option for referees to remain anonymous, and this is also very clearly specified on the journal web pages as well as in the referenced article and in a more comprehensive review article (Pöschl, 2012 as referenced and linked below). Thus, it would indeed be wrong to claim “complete transparency” for the open peer review process for ACP, but it is also wrong to insinuate that ACP or the cited article would have raised this claim. Please discard or correct the erroneous statement accordingly. Moreover, please complement or replace the reference to Pöschl 2004 by a reference to Pöschl 2012, because the latter is more comprehensive and up-to.date:
  Pöschl U (2012) Multi-stage open peer review: scientific evaluation integrating the strengths of traditional peer review with the virtues of transparency and self-regulation. *Front. Comput. Neurosci.* **6**:33. doi: 10.3389/fncom.2012.00033
http://journal.frontiersin.org/article/10.3389/fncom.2012.00033/abstract 
2. Correction of Erroneous Statement: “It could be argued that synchronous review occurs, yet *ACP* does not consider papers posted at *ACPD* to be “published.” Because these papers are not considered published, their review is not synchronous.” 
  Neither the ACP journal web pages nor the articles providing an authoritative description of the ACP open peer review (Pöschl, 2004 & 2012) have ever indicated that the papers posted in the ACP discussion forum, ACPD, would not be “published”. Well on the contrary, all relevant journal web pages emphasize explicitly that the discussion papers posted and reviewed/discussed in ACPD are publications with permanent public accessibility, archiving and citability. Also my articles explaining the concepts of interactive open access publishing and multi-stage open peer review (Pöschl, 2004 and 2014) specify and emphasize that the discussion papers published in ACPD - as well as in the fifteen interactive open access sister journals of the European Geosciences Union (EGU) - are indeed publications. Thus, it is inappropriate to insinuate the opposite. Please discard or correct the erroneous statement accordingly.
http://www.egu.eu/about/statements/position-statement-on-the-status-of-discussion-papers-published-in-egu-interactive-open-access-journals/ 
http://www.atmospheric-chemistry-and-physics.net/general_information/publication_policy.html 
http://www.atmospheric-chemistry-and-physics.net/review/review_process_and_interactive_public_discussion.html 
http://www.atmospheric-chemistry-and-physics.net/general_information/faq.html 
http://www.ingentaconnect.com/content/alpsp/lp/2004/00000017/00000002/art00005 
http://journal.frontiersin.org/article/10.3389/fncom.2012.00033/abstract  
3. General Comment and Suggestion:
  I understand and respect that the author prefers a fully transparent form of open peer review as implemented by *F1000Research*. However, *F1000Research *is a relatively recent follow-up on a series of earlier initiatives that have been pursuing open peer review and are reaching back into the last century. For example, see the British Medical Journal (BMJ), the Electronic Transactions on Artificial Intelligence (ETAI), the Journal of Interactive Media in Education (JIME) and other journals with open peer review as referenced in the recent “research topic” (special issue) on open evaluation (including open peer review) in the open access journal Frontiers in Computational Neuroscience: 
http://journal.frontiersin.org/researchtopic/137  
  As detailed in my contribution to that collection of articles (Pöschl 2012) and confirmed by independent studies referenced/linked below, ACP and its sister journals are by most standards of scientific publishing more successful and more efficient than comparable journals with traditional or alternative forms of peer review: 
http://www.atmospheric-chemistry-and-physics.net/pr_acp_is_interactive_open_access_publishing_able_to_identify_high_impact_submissions.pdf 
http://www.biomedcentral.com/1471-2288/13/74 
  Overall, I see no well-founded basis for the claim that the particular form open peer review practiced by *F1000Research *would deserve the attribute “gold standard of open peer review”, and I would suggest to substantiate or drop this postulate.

I have read this submission. I believe that I have an appropriate level of expertise to confirm that it is of an acceptable scientific standard, however I have significant reservations, as outlined above.

# Peer review: Referee response for version 1

**DOI:** 10.5256/f1000research.6426.r7270

**Published:** 2015-01-12

**Authors:** Scott Walter

**Affiliations:** 1 John T. Richardson Library, DePaul University, Chicago, IL, USA

The study applies the tools of content analysis to the stated editorial policies and procedures of a small number of journals in STEM disciplines adhering (to a greater or lesser degree) to the principles of open peer review. To promote replicability of the study and/or comparison between the set of journals included in the current study and other sets of journals, the study might be revised to include a table documenting the degree to which each journal under consideration did or did not meet the stated OPR principles. While the study is limited to review of relatively new publications and publication models in a defined field, it might also be revised to suggest future research opportunities, e.g., the degree to which OPR principles are employed in more established journals, the degree to which OPR experiments are being explored in other fields.

I have read this submission. I believe that I have an appropriate level of expertise to confirm that it is of an acceptable scientific standard.

# Response

**Published:** 2015-01-25

**Authors:** Emily Ford

**Affiliations:** Portland State University, USA

Thank you for your comments and feedback. The suggestion of a table, I think, will help to clearly communication these observations, so a revision will include one. One of the things that I have struggled with is how to represent what might be happening in other disciplinary communities. From what I have found, OPR seems to have started in STEM disciplines. I'm finding it difficult to wrap my thoughts and ideas around OPR in the humanities since humanities publishing is also changing with new ways to express scholarship, aka the digital humanities. In this way it is easier (for me) to begin observing and evaluating OPR in STEM publications. 

Again, thank you for your thoughts and comments, and I look forward to more comments after I re-submit this article.

# Peer review: Referee response for version 1

**DOI:** 10.5256/f1000research.6426.r7269

**Published:** 2015-01-12

**Authors:** Peter Binfield

**Affiliations:** 1 PeerJ, San Francisco, CA, USA

Thank you for an interesting article which overviews an emerging form of publication / peer review

**General Comments:**

At an early point, the author defines OPR as simply naming the authors and reviewers ( *“any scholarly review mechanism providing disclosure of author and referee identities to one another at any point during the peer review or publication process”*), but then goes on (e.g. in the 2 ^nd^ half of para 1 of *“Open Peer Review”*) to discuss it as if it also includes making those comments publicly available at the time of publication (something which is certainly considered part of OPR by many people). Therefore, the author either needs to expand their definition, or they need to word their commentary differently.

For example, when the author makes statements that:

*"More broadly speaking, OPR provides the scholarly community an insight into author/referee conversations during the review process."*

They are implying that the community can read the comments upon publication. This is not the case  when using the narrow definition which the author has adopted (which is stated as *“any scholarly review mechanism providing disclosure of author and referee identities to one another at any point during the peer review or publication process”* and thus does not explicitly include the possibility that peer-review comments will be made public upon publication).

I suggest that the author clearly defines what they mean as OPR for the purposes of this article

Note: It is interesting that all the studied journals are OA – is that worth a discussion? Is OA more naturally favorable to OPR? Are there any subscription journals operating OPR? If not, why not?

**Edits:**

- *“have approached is as supplementary” *should read *“have approached it as supplementary”*
- PLoS One should be written PLOS ONE throughout

- *"individuals may publish in PeerJ a dedicated number of times per year"* edit to: *“individuals may publish in PeerJ a specified number of times per year”*

- *”peer review of assigned articles to be completed by at least one reviewer,”* change to: *“peer review of assigned articles to be completed by at least two reviewers,”*

- Grammar needs fixing in: *“In addition to reviews provided each work by two or three designated expert referees“*

 

**Notes:**

- *"By publishing research from multiple disciplines, the journal boasts “PLoS One” *use of the word ‘boasts’ seems unnecessarily emotional here.
- *“The first implementation of OPR occurred at Atmospheric Chemistry and Physics with its launch in 2001” *This seems unlikely. Is there a reference? The article then says: *"Perhaps the oldest of the open peer reviewed publications is Atmospheric Chemistry and Physics”*. So which is it? The oldest? Or ‘perhaps’ the oldest?
- *”In addition to PLoS One’s version of signed review, the journal enables crowd-sourced review, which occurs post-publication.”* Although I note the authors definition of *“crowd sourced review”* (their bullet 5 in Methods), it is worth saying that at the time this article was written, PLOS ONE did not (and still doesn’t, I believe) regard (or promote) their commenting facility as a form of review. Instead they regard it as a way to enable “post publication commentary”. Same goes for PeerJ.
- *"One unique aspect of this crowd-sourced review process is that PLoS One surfaces any media coverage of published articles by linking to them"* This isn't necessarily unique, PeerJ does this as well via the ability for any user to “add link”. Also, many other journals do this via their article level metrics provision.
- *”PeerJ is an individual membership-based publisher in the biological and medical sciences.”* At the time this article was written this was correct. However, since that time, the journal changed the description of its model to “publication plans” rather than “memberships”
- *”All works published by PeerJ are licensed with a Creative Commons Attribution 3.0 Unported license” *Correct at the time the article was written. However, since that time the journal has upgraded to CC BY 4.0
- *”Based on information from PeerJ’s website, it is unclear whether author and reviewer identities are disclosed to one another during the review process.”* I can clarify this – reviewers are encouraged to name themselves (~40% choose to do so). If they do so, then the authors are first made aware of their identities in the decision letter that includes the comments of that reviewer.
- *”I examined four journals that boast open peer review processes, PLoS One,”*. Actually, I am not sure that PLOS ONE would claim this fact (and the author themselves observes that very few elements of OPR are provided by PLOS ONE)
- *”whereas F1000Resarch and PLoS One require referees to disclose their identities”.* This is not the case for PLOS ONE (unless something has changed which I am not aware of). The default for PLOS ONE reviewers is to remain anonymous.

I have read this submission. I believe that I have an appropriate level of expertise to confirm that it is of an acceptable scientific standard, however I have significant reservations, as outlined above.

# Response

**Published:** 2015-01-25

**Authors:** Emily Ford

**Affiliations:** Portland State University, USA

Thank you for your thoughtful comments. Your working knowledge of both PeerJ and PLOS ONE will benefit this article's revision, and I appreciate you offering this knowledge in your review.

Regarding your note about OPR and the overlap with OA journals: yes, OA journals are generally more sympathetic and willing to try OPR. Since you ask it is clear to me that I have not adequately discussed this overlap in the article, which I will be sure to in a revision. 

Thank you again for your comments.
